# The Relationship between the Oral Microbiota and Metabolic Syndrome

**DOI:** 10.3390/biomedicines11010003

**Published:** 2022-12-20

**Authors:** Yvonne Prince, Glenda M. Davison, Saarah F. G. Davids, Rajiv T. Erasmus, Andre P. Kengne, Lisa M. Graham, Shanel Raghubeer, Tandi E. Matsha

**Affiliations:** 1 SAMRC/CPUT/Cardiometabolic Health Research Unit, Department of Biomedical Sciences, Faculty of Health and Wellness Sciences, Cape Peninsula University of Technology, Bellville 7530, South Africa; 2 Department of Pathology, Faculty of Medicine and Health Sciences, Stellenbosch University, Cape Town 7505, South Africa; 3 Non-Communicable Diseases Research Unit, South African Medical Research Council, Cape Town 7505, South Africa; 4 Sefako Makgatho Health Sciences University, Ga-Rankuwa 0208, South Africa

**Keywords:** metabolic syndrome, oral microbiota, subgingival plaque, rDNA, oral microbiota, metabolic syndrome, South Africa, diabetes, cardiovascular disease

## Abstract

The oral microbiota plays a crucial role in both systemic inflammation and metabolic syndrome (MetS), which is characterised by low-grade inflammation. Studies have analysed the gut microbiota using stool specimens from subjects with MetS; however, the etiological role of the oral microbiota in the development of MetS is still uncertain. We investigated the oral microbiota of 128 subgingival plaque samples from a South African cohort with and without MetS. After a comprehensive analysis of the oral microbiota, we observed a significant increase in Gram-positive aerobic and anaerobic microbiota in those with MetS. We observed an abundance of Actinomyces, Corynebacterium, and Fusobacterium genera in the MetS group, which differed significantly from previous studies, which found *Granulicatella* to be enriched in MetS. To further assess the impact of the metabolic parameters (FBG, Waist C, HDL, TGs, and BP) on the oral microbiota, we calculated the odds ratio (ORs) for significant oral microbiota identified between the MetS groups. We found that different species were associated with at least four MetS risk factors. This study has shown that the oral microbiota is disrupted in MetS and may promote inflammation providing a gateway to other systemic diseases, including diabetes and cardiovascular diseases.

## 1. Background

Metabolic syndrome (MetS), known to cause low-grade inflammation, consists of a cluster of risk factors that can predispose individuals to cardiovascular disease (CVD) and type 2 diabetes mellitus (T2DM) [1]. The definition of MetS is based on the 2009 JIS (The Joint Interim Statement) criteria and includes any three of the following risk factors: central obesity, dyslipidaemia (raised triglycerides (TGs) and decreased levels of high-density lipoprotein (HDL) cholesterol, hyperglycaemia, and hypertension [2]. MetS has become a public health concern globally and is largely attributed to increasing obesity and lifestyle changes [3].

Previous studies have confirmed that the oral microbiota plays a vital role in both local and systemic inflammation and disease [4] and harbours over 1000 different species of microorganisms [5,6]. Indeed, the presence and preservation of commensal microbiota are essential in maintaining a healthy oral environment and preventing opportunistic pathogenic colonisation. These microorganisms play a role in human physiological status, including the properties of the innate and adaptive immune system, host metabolism, and genotype [7]. The oral microbiota has further been associated with various oral diseases, the most common being gingivitis and periodontitis. Recently, periodontitis has been associated with systemic diseases, such as coronary heart disease and type 2 diabetes mellitus [8,9,10].

The oral pathogen *Porphyromonas gingivalis* has been shown to induce systemic inflammation and metabolic disorders in mice [11]. These findings suggest that oral microbiota may cause low-grade inflammation in humans leading to the development of MetS. Given that the oral microbiome may be an important etiological agent in the development of systemic disease, its role in the development of MetS is important. Most previous studies have been performed on the gut microbiota using stool samples from mice [8]. In this study, we aimed to investigate and analyse the oral microbiota, using plaque samples from individuals with risk factors for MetS in order to characterise and identify microbiota signatures that are associated with MetS.

## 2. Methods and Materials

### 2.1. Study Subjects and Sample Collection

This was a case-controlled study conducted on a South African population in the Western Cape, Cape Town. A total of 128 subjects were recruited from the ongoing Vascular and Metabolic Health (VMH) study, which had obtained ethical approval from Stellenbosch University and the Cape Peninsula University of Technology (respectively, NHREC: REC-230 408-014, CPUT/HW-REC 2015/H01 and N14/01/003). The oral microbiota analysis received further ethical clearance from the Ethics Committee of the Cape Peninsula University of Technology (CPUT/HW-REC 2017/H31), and the study was performed according to the guidelines of the Helsinki Declaration.

### 2.2. Sample Collection

Blood samples were collected after overnight fasting. The oral glucose tolerance test (OGTT) was performed on all subjects except those receiving treatment for type 2 diabetes mellitus. All other blood tests were conducted in an ISO (International Standard) 15189 accredited laboratory (PathCare Reference Laboratory, Cape Town, South Africa) and included plasma glucose, HBA1c (Glycated haemoglobin), triglycerides (TG), low-density lipoprotein cholesterol (LDL-chol), high-density cholesterol (HDL-chol), insulinγ-Glutamyl transferase (GGT), and ultra-sensitive CRP (us-CRP). Lifestyle and clinical conditions were recorded using a questionnaire, and a written consent form was signed by all participants after all procedures had been fully explained in the language of their choice.

Anthropometric measurements, including waist circumference (WC) and blood pressure (BP), were obtained as follows: The waist (WaistC) and hip circumference (hipC) measurements were taken while subjects were in a relaxed position and were rounded off to the nearest 0.5 cm. The systolic (SBP) and diastolic (DBP) (mmHg) measurements were taken at three time points within a 5 min interval. The lowest SBP and its corresponding measurement were recorded and used for statistical analysis in this study. This procedure was performed according to (1999) World Health Organization (WHO) recommendations. Bodyweight (in kilograms) was measured using a calibrated Omron body fat meter HBF 511 digital bathroom scale and the Body Mass Index (BMI) (kg/m^2^) was calculated as the body weight (in kilograms) divided by the square of the subject’s height (in meters) and rounded off to the nearest 0.1 kg.

### 2.3. Metabolic Syndrome Classification

For this study, the JIS MetS classification was used to diagnose MetS [2]. The diagnosis was made if the participant presented with three or more risk factors, such as central obesity, hyperglycaemia, hypertriglyceridaemia, low HDL-cholesterol (dyslipidaemia), and hypertension. Central obesity was defined as a waist circumference of >90 cm [12], while a fasting blood glucose (FBG) of >56 indicated hyperglycaemia. Hypertension was identified when the systolic blood pressure measured ≥130 mmHg and diastolic measured ≥85 mmHg. Low HDL (men <1 mmol and women <1.3 mmol) and triglycerides measuring >1.7 mmol were indicative of dyslipidaemia.

### 2.4. Collection of Plaque Samples

After dental examination and assessment, four subgingival plaque samples were collected from all subjects after fasting for 12 h and without tooth brushing, food intake, or smoking. The plaque samples were collected using the wood toothpick method from both sides of the oral cavity, marked as right side and left side, similar to a previous study [13]. This was performed by inserting the device in the subgingival crevice between the first premolar and last upper premolar, according to the guidelines from the World Health Organization. The samples were stored at −80 °C immediately upon collection until further analysis was performed.

### 2.5. DNA Extraction and Quality Control

Microbial Plaque DNA was extracted from plaque samples using the Zymo Quick-DNA Fungal Bacterial Miniprep KIT (Zymo Research, Irvine, CA, USA), and extraction was performed according to the manufacturer’s protocol. DNA quality and quantity were determined using a NanoDrop ND-1000 spectrophotometer (Thermo Fisher Scientific, Waltham, MA, USA). The Qubit 4.0 Fluorometer was used to quantify the metagenomic DNA (mgDNA) using the Qubit dsDNA HS assay kit according to the manufacturer’s protocol (MAN0017455 Rev. A.0). The mgDNA purity was established using the NanoDrop ND-1000 Spectrophotometer, while the LapChip GXII was used to determine the genomic quality score following use of the DNA Reagent Kit (PerkinElmer, Waltham, MA, USA) and Genomic DNA (gDNA) Quality Control kit according to the manufacturer’s protocol (CLS140166 Rev. C; Supplementary Report). A genomic score between 0 and 5 was used, with 5 indicating high-quality gDNA.

### 2.6. Metagenomics 16S rDNA

Amplification of hypervariable regions from the polybacterial DNA samples was conducted using the Ion 16S Metagenomic Kit following the manufacturer’s protocol (MAN0010799 REV C.0). mgDNA (2 µL) was used to amplify the target regions across 25 cycles with 2 primer pools using the SimpliAmp Thermal Cycler (Thermo Fisher Scientific, Waltham, MA, USA). After verification of amplification (polymerase chain reaction), primer 1 (V2-4-8) and primer 2 (V3-6-7-9) pools were combined for each sample. Purification was conducted and eluted using Agencourt AMPure XP reagent and 15 µL of nuclease-free water. The Qubit 1x dsDNA HS assay kit was used to quantify the purified amplicons on the Qubit 4.0 Fluorometer following the manufacturer’s protocol (MAN001 7455 Rev. A.0).

### 2.7. Library Preparation

NEXTflex DNA Sequencing Kit was used to prepare the library, of which 100 ng of the pooled amplification product was used for each sample following the v 15.12 Bio Scientific Corporation protocol. The LabChip GXII Touch (PerkinElmer, Waltham, MA, USA) was used for library fragmentation size distribution, with the X-mark chip and HT DNA NGS 3K reagent kit according to the manufacturer’s protocol (CLS145098 Rev. E).

### 2.8. Template Preparation, Enrichment, Sequencing, and Analysis

Library dilution was performed by targeting a 10 pM concentration. Thereafter, the diluted 16S barcoded libraries were combined in equimolar amounts for template preparation using the Ion 510, Ion 520, and Ion 530 Chef Kit. Briefly, 25 µL of the pooled library was loaded on the Ion Chef liquid handler with reagents, solutions, and supplies according to the manufacturer’s protocol (MAN001 6854, REV.C.0). The Ion 530 Chip was used to load the enriched template-positive ion sphere particles onto the chip. The Ion S5 Gene Studio with the Ion S5 Sequencing Solutions and Sequencing Reagents Kit was used to run massive parallel sequencing according to the protocol (MAN0016854, REVC.0). The Torrent Suite software (v 5.12.0) was then used for flow space calibration, and BaseCaller analyses were performed with default analysis parameters. Raw sequence data and taxonomy assignment were performed as previously described [14].

### 2.9. Statistical Analysis

The software SPSS v.26 (IBM Corp, 2019) was used for data analysis. The results were reported as mean (standard deviation), median (25th and 75th percentiles), and count (percentages). For comparison, analysis of variance test (ANOVA) and Kruskal Wallis were used for numerical variables, while the chi-square test of association was used for categorical variables. Microbiome data was presented in terms of relative abundance percent for the genus, and species comprising ≤1% of the total abundance were grouped as “other”. The independent *t-*test was used to determine statistically significant differences in the relative percent abundance between cases and controls for the genus or species. Multivariate logistic regression models were used to assess the association between microorganisms present in MetS, periodontitis, and bleeding and the adjusted odds ratios (OR). A *p*-value < 0.05 was used to characterise statistically significant results. Alpha diversity was performed using Chao1, Shannon, and Simpson indices to determine species richness and how many species were present in our oral microbial samples. Emperor (v0.9.60) was used to plot the principal coordinate plot (PCO) using transformed OUT counts to determine beta analysis. Bray–Curtis calculation was used to determine the compositional dissimilarities between MetS and subjects without MetS. QIIME 2 (Quantitative Insight Into Microbial Ecology) database was used to sequence raw data, and taxonomy assignments were performed with specific software, which groups sequences of very high similarity (97%), picks Operational Taxonomic Units (OTUs), and assigns taxonomic identities based on comparisons to sequences from the reference database.

## 3. Results

**Figure 1:**
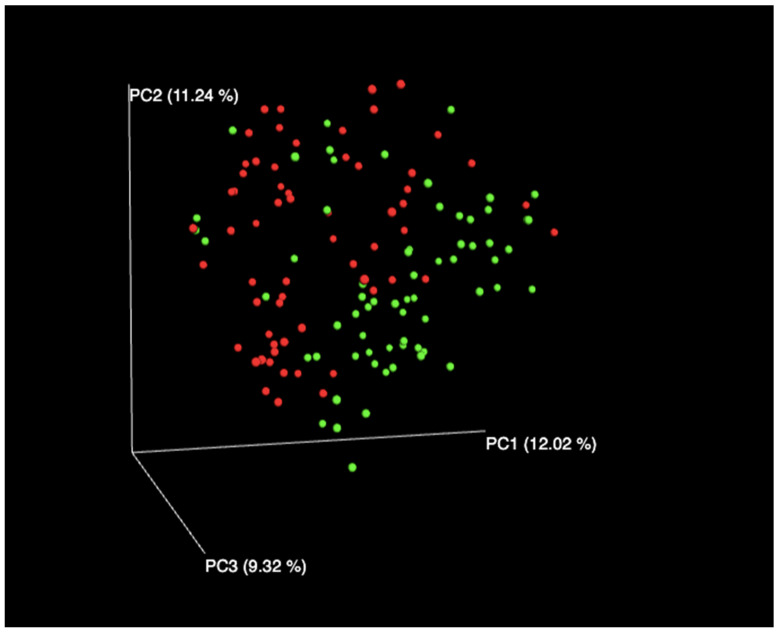
Beta diversity comparisons of microbial communities in subjects with MetS and subjects without MetS. MetS positive (red) and MetS negative (green) are shown to determine Bray–Curtis distances.

We performed 16S rDNA gene sequencing using subgingival plaque samples from 128 participants, of which 62 (48%) subjects were diagnosed with MetS. This was performed to determine and characterise the oral microbiota of subjects with and without MetS. As expected, those with MetS had significantly higher BMI, waist and hip circumferences, diastolic blood pressure, and increased hyperglycaemia and triglyceride parameters. The inflammatory marker CRP was also significantly higher in subjects with MetS (Table 1). Based on the Chao index, the alpha diversity was increased in subjects with MetS as compared with subjects without MetS (Table 2). Regarding Simpson diversity, low diversity was seen in the species communities between the two groups (Table 2). Beta diversity indicated a 24% dissimilarity in species populations between subjects with MetS and subjects without MetS (Figure 1).

A significantly higher percentage of *Actinomyces dentalis* (*p* < 0.001), *Actinomyces naeslundii* (*p* < 0.001), *Actinomyces viscosus* (*p* = 0.021), *Corynebacterium matruchotii* (*p* < 0.001), *Leptotrichia buccalis* (*p* = 0.007), and *Streptococcus sanguinis* (*p* < 0.001) were seen in MetS subjects, while lower percentages of *Actinomyces odontolyticus* (*p* = 0.005), *Campylobacter gracilis* (*p* = 0.002), *Fusobacterium canifelinum* (*p* = 0.001), *Fusobacterium nucleatum* (*p* < 0.001), *Fusobacterium periodonticum* (*p* = 0.022), *Haemophilus parainfluenzae* (*p* < 0.001), and *Veillonella rogosae* (*p* = 0.007) were observed in subjects without MetS (Table 3).

Although there was no significant difference in the incidence of periodontal disease or bleeding between those with and without MetS, *Fusobacterium nucleatum* (*p* = 0.001), *Neisseria flavescens* (*p* = 0.036), and *Veillonella rogosae* (*p* = 0.037) were increased in those with gingival bleeding, while *Granulicatella adiacens* (*p* = 0.016), *Selenomonas noxia* (*p* = 0.01), and *Streptococcus sanguinis* (*p* = 0.007) were decreased in subjects with MetS (Table 3).

To assess the impact of metabolic parameters (FBG, Waist-C, HDL, TGs, and BP) on the oral microbiota, correlations between the genus, species, and oral microbiota were performed (Supplementary Table S1). The results revealed that *Actinomyces dentalis, Actinomyces naeslundii, Actinomyces odontolyticus*, *Actinomyces viscosus*, *Campylobacter gracilis, Corynebacterium matruchotii, Fusobacterium canifelinum, Fusobacterium nucleatum, Fusobacterium periodonticum*, *Haemophilus parinfluenzae*, *Leptotrichia bucallis*, *Prevotella pellens*, *Streptococcus sanguinis*, and *Veilonella ragosae* were all positively correlated with an increased waist circumference of >90 cm (all *p* ≤ 0.042), while *Actinomyces dentalis*, *Actinomyces naeslundii*, *Actinomyces odontolyticus, Campylobacter gracilis, Corynebacterium matruchotii, Fusobacterium canifelinum*, *Fusobacterium nucleatum*, *Granulicatella adiacens*, *Haemophilus parinflenazae*, *Leptotrichia bucallis*, *Leptotrichia genomosp*, *Mannheima varigena*, *Selenomas noxia*, *Streptococcus sanguinis*, *Veilonella parvula*, and *Veilonella ragosae* (all *p* ≤ 0.045) correlated with a fasting blood glucose of >5.6 mmol/L.

Furthermore, a significant positive correlation was observed between triglycerides (>1.7 mmol/L) and *Actinomyces naeslundii*, *Actinomyces odontolyticus*, *Aggregatibacter segnis*, *Corynebacterium matruchotii*, *Fusobacterium canifelinum*, *Fusobacterium nucleatum*, *Fusobacterium periodonticum*, and *Streptococcus sanguinis* (all *p* ≤ 0.015). Positive associations between HDL (men ≤ 1 mmol/L and women ≤ 1.3 mmol/L) and *Actinomyces naeslundii*, *Actinomyces odontolyticus*, *Aggregatibacter segnis*, *Corynebacterium matruchotii*, *Fusobacterium periodonticum*, and *Haemophilus parinfluenzae* (all *p* ≤ 0.038) were also recorded (Supplementary Table S1).

*Actinomyces dentalis*, *Actinomyces naeslundii*, *Corynebacterium matruchotii*, *Fusobacterium canifelinum*, *Fusobacterium nucleatum*, *Leptotrichia genomosp*, *Leptotrichia wadei*, *Prevotella oris*, *Streptococcus gordonii*, *Streptococcus sanguinis*, and *Veilonella parvula* (all *p* ≤ 0.045) were positively correlated with age, while the inflammatory marker CRP was significantly associated with *Actinomyces odontolyticus*, *Fusobacterium periodonticum*, *Haemophilus parinfluenzae*, *Mannheima varigena*, *Prevotella histicola*, and *Prevotella oulorum* (all *p* ≤ 0.047; Supplementary Table S1).

To further assess the impact of MetS on the oral microbiota, we performed a multivariate logistic regression on genus and species (Supplementary Table S2). The species that remained significant throughout the odds ratio was *Campylobacter gracilis* (OR 0.29, 95% CI 0.12; 0.68, *p* = 0.005), although reduced abundance was observed.

## 4. Discussion

The literature states that the presence of periodontal pathogens and their metabolic by-products may influence the immune response beyond the oral cavity, thus promoting the development of systemic conditions. As MetS is characterised by low-grade inflammation, this study aimed to investigate and report on oral pathogens associated with subjects with and without MetS. In this study involving 128 participants, 62 subjects met the criteria of MetS, and 16S rDNA analysis revealed a significant difference in the oral microbiota between the two groups. Those who met the criteria for MetS had a significantly enriched abundance of Gram-positive aerobic and anaerobic microorganisms (20%), while Gram-negative bacteria (gnb) (18%) were less abundant.

In the MetS subjects, the most dominant Gram-positive genera were the Actinomyces and Corynebacterium (Gram-positive), while *Haemophilus parainfluenzae* was more abundant among the healthy controls. This finding differs from other research. For instance, in a comprehensive analysis of the oral microbiota genera, Granulicatella and Neisseria were found to be abundant in subjects with MetS, while Peptococcus was abundant in those without MetS [8]. *Actinomyces* spp. are Gram-positive pleomorphic bacteria (gpb) that are part of the normal flora of the oral cavity and do not normally cause disease if they are confined to the surface of the mucosa. However, when mucosal integrity is compromised, and defence mechanisms are disrupted, these bacteria can settle on deep periodontal tissues and may cause pathologic reactions and progress into periodontal disease [15]. Although the presence of bleeding was not significant, *Actinomyces naeslundii* was the most predominant Actinomyces species in both MetS and those with gingival bleeding. This is in accordance with previous studies which reported *Actinomyces naeslundii* as abundant in individuals with gingival bleeding [16]. *Actinomyces naeslundii* forms part of the “early colonisers” and forms the basis for colonisation of the sulcus with other periodontitis-associated microbiota. This was confirmed recently when *Actinomyces naeslundii* was reported to induce horizontal alveolar bone loss similar to that caused by periodontal pathogenic bacteria, *Porphoromonas gingivalis,* which is the main etiological factor in *periodontal diseases* [17].

Periodontal disease is considered an inflammatory disease and has been linked with systemic diseases, such as diabetes and metabolic syndrome [18]. It has been hypothesised that the presence of these oral pathogens may cause systemic oxidative stress and may serve as a potential marker for both periodontitis and MetS [19]. Research has suggested that the Actinomyces species are present as polymicrobial flora and, therefore, co-aggregates with *Eikenella corrodens* from the green-complex have been observed [20].

The second most dominant gpb present in our MetS subjects was *Corynebacterium matruchotii.* This oral microbiota is present in human biofilm formation and has been associated with oral lichen planus (OLP), which is a common chronic inflammatory disease affecting the oral mucosa. In a previous study comparing host cell gene expression profiles with oral microbial profiles within patients with OLP and healthy individuals, researchers found that Corynebacterium matruchotii, *Fusobacterium periodonticum*, and other species were capable of activating the Hepatocyte nuclear factor-alpha (*HNF4A*) gene network, which mediates mucosal inflammatory processes [21]. As we also observed an abundance of this bacteria, it strongly suggests that these changes could be associated with inflammation.

Although in less abundance than those without MetS, the third most dominant genera in the MetS participants was Fusobacteria. The dominant species, *Fusobacterium nucleatum,* which is normally present as a commensal of the human oral activity, is an opportunistic orange complex anaerobic Gram-negative bacterium and is the most dominant species present in periodontal disease. In the present study, although the presence of this bacteria was lower in those with MetS, it was significantly increased in those with gingival bleeding. Previous studies have confirmed its role in other chronic inflammatory conditions and have suggested that its presence could adversely influence clinical outcomes [22]. Although *Fusobacterium nucleatum* normally presents as an oral flora, it may be of benefit due to its ability to encourage the production of antimicrobial peptides in gingival epithelial cells [23,24]. Therefore, a reduction in this bacteria, as observed in our study, may lead to a disruption in the overall balance of the oral biome leading to dysbiosis and abnormal immune responses.

The relationship between *Fusobacterium nucleatum* and the adaptive immune response is unclear, and it is thought that in healthy individuals, the immune system and the normal oral microbiota exist in synergy. However, when the oral microbiome is disrupted, T-cells, B-cells, and innate immune cells become activated, which can lead to systemic inflammation. To study this, T-cell and antibody responses to FadA and Td92 (*Fusobacterium nucleatum* specific antigens), respectively, were investigated in individuals with chronic periodontitis (CP) and healthy controls. The results demonstrated that in both groups, there was an increase in antibodies specific to the FadA antigen, together with an increase in CD4+ specific T-cells. The production of the cytokines IFNγ and IL-10 was also elevated. Although there was no difference between the healthy and CP groups, the numbers in this study were small, and those with CP had an increased response [25]. This research clearly demonstrated that the relationship between the immune response and oral bacteria is important and may lead to systemic inflammation, which may play a role in the development of metabolic syndrome.

Systemic oxidative stress may also be associated with periodontitis and MetS. Increased inflammatory markers, such as cytokines and oxidative stress markers, due to periodontitis may lead to reduced or inactive insulin sensitivity, which is considered a significant event in the development of MetS. MetS may have various definitions or classifications in the literature, but the consistent pathophysiology of MetS is insulin resistance and obesity [26]. Studies have found that insulin resistance and inflammation increases with age, and therefore, older people are at higher risk of developing CVDs [27]. This was confirmed by our findings, as those with MetS were significantly older and displayed increased CRP levels, which is a marker of inflammation.

*Actinomyces dentalis*, *Actinomyces naeslundii, Corynebacterium matruchotii*, *Fusobacterium canifelinum*, *Fusobacterium nucleatum*, *Prevotella oris*, and *Streptococcus sanguinis* displayed a significant positive correlation when the presence of oral microbiota was correlated with age and insulin resistance, using multivariate analyses. These findings support previous studies in which the supragingival biofilms of healthy periodontium in subjects over 60 years old were investigated. Researchers found a predominance of Gram-positive, aerobic bacteria and relatively fewer Gram-negative anaerobes [28]. The results demonstrated that the levels of Actinomyces species (blue complex), particularly *Actinomyces naeslundii* and *Actinomyces oris,* were significantly higher in older individuals. Our findings have further been supported by research indicating the diversity of the oral microbiota within different oral niches from older adults without root caries or periodontitis. These included species such as *Streptococcus oralis*, *Veillonella atypica*, *Streptococcus parasanguinis,* and *Fusobacterium nucleatum* [29]. Similar to our study, an abundance of *Fusobacterium nucleatum* was reported. This study also highlighted the importance of differing techniques, sample sites, and geographical areas in contributing to the controversies among researchers.

Multivariate analysis results indicated that the only bacteria that remained significant throughout was *Campylobacter gracilis.* Although the presence of *Campylobacter gracilis* remained low in MetS, its presence in the oral microbiota may signify different stages of periodontal disease [30,31]. Other authors have determined that the percentage or proportion of species within the genus Campylobacter is an important marker for periodontitis progression. Henne et al. hypothesised that the progression of periodontal disease can be predicted by the presence of three different species. These included *Campylobacter rectus* (higher abundance) and *Campylobacter concisus* (lower abundance), while the presence of *Campylobacter gracilis* is associated with intermediate progression [32]. Although oral microbiota from the yellow, green, purple, and blue complexes are usually associated with normal periodontal health, compelling evidence suggests that these bacteria may be associated with periodontal disease and systemic diseases [33,34,35].

The present study had several limitations, including a small sample size and significantly fewer males in comparison to females. In addition, plaque samples were collected at only two sites within the oral cavity and thus may not adequately represent the microbial profiles of the entire microbiota. Furthermore, this was a cross-sectional study and further longitudinal studies are required to analyse how changes in the oral microbiota may lead to disease progression. Another limitation of this study is presented by the diversity differences of phylum, genus, and/or species between individuals with and without MetS using an end-point PCR platform. For future studies, we suggest validating these samples in a larger cohort.

Other studies have confirmed that non-alcoholic fatty liver disease (NAFLD) has been associated with MetS and may be a risk factor for developing diabetes, neoplasia, and cardiovascular disease, as well as influencing obesity-related morbidity and mortality [36]. Furthermore, obesity-associated inflammation is characterised by the infiltration of macrophages into adipose tissue, which is a source of stem cells. Associations between the consequential release of inflammatory cytokines and growth factors, including stem cell growth factor-beta (SCGF-b), granulocyte and macrophage colony-stimulating factor (GM-CSF), and macrophage colony-stimulating factor (M-CSF), have been described. These processes have further been linked with insulin resistance and NAFLD [36]. The relationship between NAFLD (and secreted molecules) with the oral microbiota was not analysed in this study and should be included in follow-up research.

Nutritional status, dietary intake, and exercise were not monitored or accessed in this study. It is well documented that exercise and appropriate nutrition can reverse both disruptions of the microbiome and MetS. Therefore, it has been recommended that lifestyle interventions, such as an improved diet, be initiated for individuals with periodontal disease [36]. Future work should therefore include an analysis of these interventions and their effects on the oral biota.

Despite these limitations, the findings of this study suggest that the variation and distribution of the oral pathogens in MetS may be associated with chronic inflammation and may provide a gateway to systemic disorders. The most abundant genera observed in this cohort of MetS individuals were *Actinomyces, Corynebacterium,* and *Fusobacterium*, which differs from several other reports [8] and may be due to the population group and both sampling and analytical techniques used. Despite variation in the literature, our findings support the theory that disruptions in the oral biome can be associated with both local and systemic inflammation.

## 5. Conclusions

In this study, using 16S rDNA gene sequencing to examine the oral microbiota of individuals with MetS, changes in the abundance and distribution of microorganisms have been demonstrated, with the most significant being increases in gram-positive aerobic and anaerobic microbes. Although these are preliminary observations, these findings may be important in influencing both the innate and adaptive immune response; therefore, further studies are warranted.

## Figures and Tables

**Table 1:** General classification of participants according to the JIS classification.

	JIS = No	JIS = Yes	*p*-Value
	(*n* = 66)	(*n* = 62)	
Age (years)	44.24 (14.55)	49.98 (10.45)	0.012
BMI (kg/m^2^)	25.55 (6.12)	37.82 (8.72)	<0.001
Waist-C (cm)	80.70 (12.63)	110.5 (14.69)	<0.001
Hip (cm)	92.07 (13.00)	119.3 (18.03)	<0.001
SBP (mmHg)	126.1 (23.46)	133.3 (22.41)	0.079
DBP (mmHg)	81.80 (14.60)	87.24 (12.32)	0.025
Glucose Fasting Blood (mmol/L)	4.90 (4.50; 5.40)	7.30 (6.15; 11.05)	<0.001
Glucose2HRsPostPrandial (mmol/L)	6.95 (5.20; 8.80)	11.80 (9.90; 15.50)	<0.001
GlycatedHBHbA1cTrial (%)	5.50 (5.28; 5.93)	7.30 (6.58; 9.30)	<0.001
Insulin Fasting (mIU/L)	5.00 (3.40; 8.40)	15.90 (8.15; 22.40)	<0.001
Insulin120Minutes (mIU/L)	32.10 (19.18; 64.15)	67.50 (39.10; 110.30)	0.003
TriglyceridesS (mmol/L)	1.03 (0.77; 1.32)	1.63 (1.25; 2.19)	<0.001
LDL Cholesterol (mmol/L)	2.65 (2.20; 3.23)	3.40 (3.00; 4.15)	<0.001
Cholesterol HDLS (mmol/L)	1.50 (1.20; 1.80)	5.20 (4.70; 6.13)	<0.001
CholesterolS (mmol/L)	4.90 (4.10; 5.53)	4.90 (4.10; 5.53)	0.059
CRP (mg/L)	2.24 (1.38; 5.72)	7.19 (3.70; 15.18)	<0.001
Gamma GTS (IU/L)	32.50 (21.00; 54.00)	34.00 (25.75; 65.25)	0.228
Gender			0.049
Female (*n* = 93)	43 (65%)	50 (81%)	
Male (*n* = 35)	23 (35%)	12 (19%)	
Gingival bleeding			0.964
No (*n* = 44)	23 (35%)	21 (35%)	
Yes (*n* = 81)	42 (65%)	39 (65)	
Periodontitis			0.499
No (*n* = 56)	31 (48%)	25 (42%)	
Yes (*n* = 69)	34 (52%)	35 (58%)	

**Table 2:** Alpha diversity in species indices according to metabolic syndrome status.

	Metabolic Syndrome	
	No	Yes
Number of taxa	253	275
Shannon	4.207	4.239
Chao1	253	275
Simpson	0.0296	0.0297

**Table 3:** Genus and species associated with MetS and periodontal status.

	MetS			Periodontitis			Bleeding		
	No	Yes	*p*-Value	No	Yes	*p*-Value	No	Yes	*p*-Value
*Actinomyces dentalis*	1.59	2.80	<0.001	1.79	2.54	0.117	2.07	2.28	0.513
*Actinomyces naeslundii*	1.48	4.03	<0.001	2.58	2.15	0.166	2.93	1.99	0.056
*Actinomyces odontolyticus*	1.04	0.67	0.005						
*Actinomyces viscosus*	0.27	1.58	0.021						
*Aggregatibacter segnis*	2.52	1.81	0.174	2.13	2.08	0.438	1.33	2.56	0.152
*Campylobacter gracilis*	3.71	2.22	0.002	2.94	3.07	0.984	3.35	2.81	0.123
*Capnocytophaga leadbetteri*	1.34	1.01	0.219	1.02	1.23	0.607	1.12	1.14	0.802
*Corynebacterium matruchotii*	2.97	7.91	<0.001	5.94	5.07	0.281	5.72	5.31	0.706
*Fusobacterium canifelinum*	1.22	0.72	0.001	1.13	0.81	0.187			
*Fusobacterium nucleatum*	7.09	4.62	<0.001	6.23	5.68	0.447	4.49	6.78	0.001
*Fusobacterium periodonticum*	1.68	0.38	0.022	0.95	1.17	0.328	0.72	1.27	0.249
*Granulicatella adiacens*							1.13	0.69	0.016
*Haemophilus parainfluenzae*	10.10	3.94	<0.001	7.78	6.75	0.712	9.08	6.11	0.051
*Leptotrichia buccalis*	1.45	2.51	0.007	1.52	2.38	0.356	2.41	1.75	0.592
*Leptotrichia genomosp.*	0.97	1.84	0.183	1.12	1.63	0.538	1.39	1.41	0.329
*Leptotrichia hofstadii*	0.49	1.01	0.184				1.06	0.58	0.343
*Leptotrichia hongkongensis*				1.33	0.47	0.261	1.47	0.49	0.236
*Leptotrichia wadei*	1.23	1.43	0.327	1.18	1.49	0.180	1.24	1.42	0.495
*Mannheimia varigena*	3.54	2.03	0.075	2.86	2.80	0.150	2.99	2.74	0.462
*Neisseria flavescens*	1.19	0.57	0.835				0.57	1.02	0.036
Other *	38.22	40.17	0.118	41.21	41.56	0.646	38.50	40.62	0.528
*Prevotella histicola*	0.39	1.00	0.225						
*Prevotella maculosa*				1.00	0.91	0.763	1.03	0.90	0.197
*Prevotella melaninogenica*	3.71	4.13	0.405	4.09	3.92	0.514	2.94	4.62	0.124
*Prevotella oris*	0.73	1.34	0.160	1.09	1.02	0.227	1.37	0.86	0.059
*Prevotella oulorum*	1.32	0.75	0.069	1.06	1.06	0.255	1.50	0.80	0.326
*Prevotella pallens*	1.39	0.90	0.078	1.11	1.18	0.983	0.99	1.24	0.722
*Prevotella veroralis*	1.17	1.09	0.818	1.23	1.08	0.787	0.68	1.43	0.084
*Selenomonas noxia*	1.19	1.04	0.534	0.92	1.30	0.273	1.55	0.88	0.001
*Streptococcus gordonii*	1.03	0.67	0.339						
*Streptococcus mutans*				0.33	1.39	0.269	1.08	0.81	0.836
*Streptococcus sanguinis*	0.43	1.56	<0.001	1.35	0.65	0.209	1.76	0.50	0.007
*Veillonella alcalescens*	3.96	4.37	0.954	3.94	4.43	0.414	3.62	4.56	0.541
*Veillonella parvula*	1.08	0.94	0.140	1.03	1.02	0.933	0.92	1.08	0.919
*Veillonella rogosae*	1.37	0.89	0.007	1.13	1.16	0.757	0.87	1.31	0.037

* Dependent on genus/species count and/or relative abundance.

## Data Availability

The datasets generated and/or analysed during the current study are not publicly available due to the terms of consent to which participants agreed but are available from the principal investigator of the main study on reasonable request. The sequence data used to support the findings of this study have been deposited in the SRA BioProject database (accession number: PRJNA723337).

## Supplementary Materials

The following supporting information can be downloaded at: https://www.mdpi.com/article/10.3390/biomedicines11010003/s1, Supplementary Table S1: Correlation table indicating genus and species and impact of metabolic parameters; Supplementary Table S2: Odds ratio indicating genus and species vs MetS.
