# Supplementary material for: The Relationship between the Oral Microbiota and Metabolic Syndrome
Source: Biomedicines. 2022 Dec 20;11(1):3. doi: 10.3390/biomedicines11010003 (PMC9855685; doi:10.3390/biomedicines11010003)
Supplement: Supplementary file 1 [file biomedicines-11-00003-s001.zip › biomedicines-2041032-supplementary.pdf]

Supplementary Materials

The Relationship between the Oral Microbiota and Metabolic Syndrome

Yvonne Prince, Glenda M. Davison, Saarah F. G. Davids, Rajiv T. Erasmus, Andre P. Kengne, Lisa M. Graham, Shanel Raghubeer and Tandi E. Matsha

Table S1. Correlation table indicating genus and species and impact of metabolic parameters.

	Age		BMI		Waist		SBP		DBP		FBG		HBA1c		Insulin fasting		Trigs		LDL		HDL		CRP	
	r	*P*-value	r	*P*-value	r	*P*-value	r	*P*-value	r	*P*-value	r	*P*-value	r	*P*-value	r	*P*-value	r	*P*-value	r	*P*-value	r	*P*-value	r	*P*-value
Actinomyces dentalis	0.218	0.014	0.305	0.001	0.448	<0.001	0.093	0.298	0.064	0.479	0.293	0.001	0.316	<0.001	0.264	0.003	0.096	0.284	0.264	0.003	−0.144	0.112	0.080	0.372
Actinomyces naeslundii	0.200	0.024	0.445	<0.001	0.456	<0.001	−0.042	0.639	−0.104	0.246	0.300	0.001	0.261	0.003	0.410	<0.001	0.245	0.006	0.217	0.015	−0.234	0.009	0.131	0.143
Actinomyces odontolyticus	−0.023	0.803	−0.244	0.008	−0.291	0.001	−0.021	0.817	−0.073	0.431	−0.240	0.008	−0.292	0.001	−0.331	<0.001	−0.190	0.038	−0.310	0.001	0.250	0.006	−0.187	0.041
Actinomyces viscosus	−0.049	0.618	0.301	0.002	0.284	0.003	−0.131	0.179	−0.026	0.789	0.113	0.248	0.127	0.191	0.306	0.001	0.089	0.360	0.184	0.059	−0.169	0.086	0.077	0.428
Aggregatibacter segnis	−0.095	0.313	−0.118	0.213	−0.169	0.073	0.128	0.172	0.075	0.423	−0.096	0.307	−0.165	0.078	−0.246	0.008	−0.205	0.029	−0.163	0.084	0.215	0.022	−0.059	0.534
Campylobacter gracilis	−0.143	0.109	−0.072	0.424	−0.243	0.006	−0.161	0.071	−0.164	0.065	−0.328	<0.001	−0.194	0.029	−0.123	0.170	−0.172	0.055	−0.162	0.070	0.018	0.841	0.128	0.151
Capnocytophaga leadbetteri	0.013	0.886	−0.091	0.323	−0.079	0.389	0.141	0.123	0.146	0.111	−0.005	0.960	−0.015	0.867	−0.190	0.037	−0.079	0.389	0.028	0.760	0.122	0.185	−0.052	0.573
Corynebacterium matruchotii	0.339	<0.001	0.372	<0.001	0.495	<0.001	0.144	0.120	0.117	0.206	0.390	<0.001	0.394	<0.001	0.420	<0.001	0.236	0.010	0.341	<0.001	−0.192	0.038	0.116	0.210
Fusobacterium canifelinum	−0.234	0.009	−0.226	0.012	−0.305	0.001	−0.056	0.536	0.046	0.609	−0.300	0.001	−0.289	0.001	−0.243	0.007	−0.219	0.014	−0.062	0.497	0.104	0.253	−0.091	0.310
Fusobacterium nucleatum	−0.336	<0.001	−0.410	<0.001	−0.476	<0.001	−0.061	0.491	0.036	0.685	−0.299	0.001	−0.350	<0.001	−0.319	<0.001	−0.297	0.001	−0.143	0.109	0.116	0.196	−0.171	0.054
Fusobacterium periodonticum	−0.067	0.500	−0.290	0.003	−0.279	0.005	0.078	0.435	0.046	0.644	−0.080	0.423	−0.186	0.060	−0.395	<0.001	−0.239	0.015	−0.214	0.031	0.286	0.004	−0.196	0.047
Granulicatella adiacens	−0.107	0.242	−0.031	0.736	−0.118	0.199	0.031	0.738	−0.026	0.776	−0.212	0.019	−0.212	0.019	−0.022	0.814	−0.006	0.945	−0.053	0.564	−0.009	0.920	−0.076	0.408
Haemophilus parainfluenzae	−0.132	0.161	−0.192	0.043	−0.301	0.001	0.009	0.924	0.039	0.684	−0.336	<0.001	−0.393	<0.001	−0.224	0.017	−0.128	0.178	−0.134	0.157	0.250	0.008	−0.227	0.015
Leptotrichia buccalis	0.086	0.363	0.212	0.025	0.323	<0.001	0.134	0.155	0.146	0.120	0.348	<0.001	0.290	0.002	0.215	0.022	0.156	0.099	0.176	0.063	−0.010	0.915	0.079	0.402
Leptotrichia genomosp.	0.196	0.045	0.120	0.227	0.175	0.075	0.065	0.512	0.062	0.532	0.200	0.041	0.275	0.004	0.095	0.337	0.069	0.482	0.158	0.110	−0.020	0.845	0.008	0.938
Leptotrichia hofstadii	0.035	0.724	0.121	0.224	0.154	0.120	0.036	0.716	0.037	0.710	0.109	0.269	0.119	0.228	0.248	0.012	0.060	0.546	0.150	0.130	−0.127	0.203	0.102	0.303
Leptotrichia hongkongensis	0.046	0.654	0.166	0.104	0.133	0.193	−0.084	0.411	−0.102	0.317	−0.148	0.145	−0.037	0.715	0.135	0.187	0.182	0.073	0.119	0.241	−0.079	0.441	−0.046	0.652
Leptotrichia wadei	0.221	0.017	0.153	0.106	0.181	0.055	0.078	0.408	−0.011	0.906	0.032	0.732	0.210	0.024	0.155	0.099	0.065	0.489	0.359	<0.001	−0.055	0.564	0.049	0.604
Mannheimia varigena	−0.088	0.340	−0.025	0.789	−0.112	0.223	0.048	0.602	0.068	0.459	−0.187	0.040	−0.255	0.005	−0.084	0.359	−0.055	0.551	−0.053	0.568	0.029	0.751	−0.226	0.013
Neisseria flavescens	0.075	0.459	−0.043	0.672	−0.001	0.995	0.016	0.873	−0.044	0.661	0.006	0.951	−0.103	0.309	−0.025	0.805	−0.071	0.485	0.021	0.839	0.029	0.775	−0.120	0.234

Prevotella histicola	−0.047	0.650	0.198	0.055	0.173	0.094	−0.079	0.448	0.075	0.473	0.058	0.575	0.212	0.040	0.183	0.078	−0.059	0.572	0.074	0.479	−0.071	0.502	0.212	0.039
Prevotella maculosa	0.002	0.982	0.108	0.236	0.054	0.552	−0.137	0.129	−0.120	0.183	−0.057	0.526	0.049	0.589	0.133	0.144	0.062	0.498	−0.034	0.705	−0.115	0.208	0.048	0.595
Prevotella melaninogenica	0.030	0.747	−0.135	0.141	−0.101	0.270	−0.049	0.594	−0.003	0.972	0.031	0.736	−0.120	0.187	−0.134	0.143	0.047	0.610	−0.073	0.424	0.067	0.467	0.005	0.952
Prevotella oris	0.234	0.012	0.161	0.090	0.170	0.072	0.062	0.515	0.000	0.996	0.042	0.655	0.059	0.530	0.208	0.027	0.116	0.219	0.093	0.327	−0.154	0.105	0.036	0.705
Prevotella oulorum	−0.058	0.539	0.058	0.538	−0.029	0.759	−0.181	0.052	−0.240	0.009	−0.108	0.248	0.012	0.902	−0.024	0.803	−0.143	0.127	−0.009	0.925	−0.011	0.905	0.191	0.040
Prevotella pallens	0.030	0.777	−0.178	0.099	−0.217	0.042	0.056	0.605	0.014	0.897	−0.145	0.175	−0.173	0.105	−0.249	0.019	−0.042	0.694	−0.002	0.985	0.177	0.098	0.012	0.908
Prevotella veroralis	−0.036	0.702	0.081	0.396	0.036	0.706	−0.113	0.228	−0.162	0.083	0.053	0.576	0.088	0.352	0.120	0.202	0.094	0.316	−0.016	0.865	−0.150	0.112	−0.029	0.757
Selenomonas noxia	0.173	0.056	0.187	0.040	0.147	0.108	−0.015	0.873	−0.056	0.539	−0.043	0.635	0.051	0.578	0.164	0.072	−0.006	0.950	0.029	0.749	0.040	0.661	0.080	0.381
Streptococcus gordonii	−0.230	0.010	0.022	0.809	−0.083	0.366	−0.029	0.746	−0.017	0.849	−0.218	0.016	−0.155	0.087	−0.099	0.280	−0.099	0.276	0.029	0.749	0.140	0.127	−0.054	0.553
Streptococcus mutans	−0.126	0.345	−0.109	0.416	−0.149	0.263	0.001	0.994	0.104	0.439	−0.154	0.250	−0.144	0.282	−0.255	0.055	−0.029	0.827	0.284	0.032	0.084	0.533	−0.006	0.967
Streptococcus sanguinis	0.252	0.007	0.436	<0.001	0.376	<0.001	0.112	0.238	0.093	0.327	0.189	0.045	0.109	0.250	0.409	<0.001	0.269	0.004	0.213	0.024	−0.123	0.198	0.094	0.321
Veillonella alcalescens	−0.057	0.522	0.118	0.191	−0.017	0.853	−0.072	0.418	−0.071	0.425	−0.164	0.065	−0.129	0.148	0.004	0.964	−0.051	0.567	−0.051	0.570	0.025	0.783	0.001	0.993
Veillonella parvula	−0.201	0.025	−0.093	0.307	−0.148	0.101	−0.279	0.002	−0.224	0.012	−0.249	0.005	−0.222	0.013	−0.171	0.058	−0.080	0.380	−0.171	0.057	0.044	0.632	−0.033	0.715
Veillonella rogosae	−0.147	0.099	−0.100	0.266	−0.212	0.017	−0.100	0.263	−0.101	0.260	−0.244	0.006	−0.150	0.093	−0.192	0.031	−0.162	0.070	−0.013	0.886	0.087	0.337	−0.041	0.644
Mets Other	0.144	0.105	0.176	0.049	0.172	0.054	0.105	0.238	0.151	0.090	0.000	0.999	0.086	0.337	0.166	0.062	0.035	0.695	0.118	0.186	−0.026	0.772	0.158	0.075
Perid_Other	0.011	0.904	0.145	0.105	0.094	0.293	0.005	0.956	0.117	0.189	0.000	0.996	0.012	0.897	0.139	0.118	−0.005	0.954	0.092	0.305	0.006	0.950	0.173	0.051
Bleeding Other	0.013	0.888	0.131	0.142	0.082	0.357	0.018	0.842	0.129	0.147	0.017	0.850	0.032	0.719	0.137	0.125	0.010	0.910	0.100	0.263	0.001	0.994	0.156	0.079

Table S2. Odds ratio indicating genus and species vs MetS.

	Model 1		Model 2		Model 3		Model 4		Model 5		Model 6	
	**OR (95% CI)**	***P*-value**	**OR (95% CI)**	***P*-value**	**OR (95% CI)**	***P*-value**	**OR (95% CI)**	***P*-value**	**OR (95% CI)**	***P*-value**	**OR (95% CI)**	***P*-value**
Actinomyces dentalis	3.33 (1.58; 7.03)	0.002	2.14 (0.88; 5.22)	0.094	2.07 (0.85; 5.04)	0.112	2.07 (0.83; 5.13)	0.117	2.02 (0.76; 5.34)	0.156	2.00 (0.74; 5.45)	0.174
Actinomyces naeslundii	2.21 (1.24; 3.95)	0.007	1.10 (0.78; 1.55)	0.584	0.96 (0.46; 1.99)	0.906	0.89 (0.45; 1.77)	0.744	0.72 (0.35; 1.45)	0.356	0.72 (0.34; 1.51)	0.382
Actinomyces odontolyticus	0.17 (0.03; 0.82)	0.027	0.31 (0.04; 2.31)	0.252	0.28 (0.04; 2.17)	0.224	0.40 (0.05; 3.23)	0.392	0.89 (0.08; 10.08)	0.927	1.29 (0.1; 16.28)	0.843
Actinomyces viscosus	5.10 (0.54; 48.32)	0.155	1.18 (0.64; 2.17)	0.594	0.98 (0.28; 3.46)	0.979	0.92 (0.26; 3.29)	0.897	1.04 (0.31; 3.51)	0.944	1.11 (0.31; 4.00)	0.876
Aggregatibacter segnis	0.82 (0.53; 1.25)	0.354	0.93 (0.49; 1.77)	0.820	0.85 (0.44; 1.66)	0.639	0.92 (0.49; 1.75)	0.808	1.20 (0.50; 2.87)	0.679	1.22 (0.53; 2.81)	0.638
Campylobacter gracilis	0.52 (0.32; 0.85)	0.008	0.30 (0.15; 0.60)	0.001	0.30 (0.14; 0.62)	0.001	0.29 (0.14; 0.60)	0.001	0.29 (0.12; 0.70)	0.006	0.29 (0.12; 0.68)	0.005
Capnocytophaga leadbetteri	0.58 (0.22; 1.53)	0.270	0.64 (0.15; 2.66)	0.540	0.72 (0.17; 3.03)	0.654	0.65 (0.15; 2.79)	0.562	0.76 (0.17; 3.44)	0.721	0.66 (0.14; 3.07)	0.594
Corynebacterium matruchotii	1.46 (1.18; 1.80)	0.001	1.30 (1.01; 1.67)	0.042	1.29 (1.00; 1.66)	0.053	1.31 (1.01; 1.69)	0.039	1.24 (0.95; 1.63)	0.120	1.24 (0.94; 1.63)	0.131
Fusobacterium canifelinum	0.06 (0.01; 0.34)	0.002	0.12 (0.01; 1.22)	0.074	0.07 (0.01; 0.90)	0.041	0.07 (0.00; 0.92)	0.043	0.12 (0.01; 1.99)	0.139	0.14 (0.01; 2.38)	0.172
Fusobacterium nucleatum	0.42 (0.27; 0.66)	<0.001	0.67 (0.37; 1.19)	0.171	0.58 (0.31; 1.07)	0.081	0.68 (0.38; 1.22)	0.192	0.88 (0.45; 1.74)	0.723	0.91 (0.49; 1.71)	0.772
Fusobacterium periodonticum	0.16 (0.04; 0.60)	0.006	0.21 (0.04; 1.20)	0.080	0.15 (0.02; 0.98)	0.047	0.19 (0.03; 1.07)	0.060	0.27 (0.04; 1.79)	0.175	0.26 (0.04; 1.67)	0.154
Granulicatella adiacens	0.88 (0.23; 3.32)	0.849	0.57 (0.10; 3.28)	0.530	0.81 (0.13; 5.05)	0.823	0.64 (0.11; 3.67)	0.615	0.86 (0.09; 8.15)	0.893	0.76 (0.09; 6.27)	0.798
Haemophilus parainfluenzae	0.84 (0.72; 0.97)	0.019	0.87 (0.74; 1.03)	0.109	0.89 (0.76; 1.04)	0.149	0.87 (0.73; 1.03)	0.096	0.91 (0.81; 1.02)	0.107	0.90 (0.79; 1.01)	0.082
Leptotrichia buccalis	1.62 (1.01; 2.60)	0.045	1.44 (0.88; 2.36)	0.142	1.60 (0.96; 2.68)	0.073	1.46 (0.88; 2.40)	0.143	1.36 (0.76; 2.44)	0.295	1.31 (0.74; 2.31)	0.357

Leptotrichia genomosp.	2.04 (1.00; 4.14)	0.049	1.68 (0.68; 4.15)	0.262	1.58 (0.65; 3.84)	0.314	1.59 (0.65; 3.90)	0.313	1.22 (0.51; 2.95)	0.652	1.31 (0.54; 3.18)	0.554
Leptotrichia hofstadii	2.12 (0.67; 6.69)	0.202	2.75 (0.42; 18.15)	0.292	3.01 (0.45; 20.15)	0.255	2.61 (0.40; 16.99)	0.315	1.62 (0.40; 6.50)	0.497	1.57 (0.40; 6.14)	0.515
Leptotrichia hongkongensis	1.02 (0.63; 1.67)	0.922	0.69 (0.39; 1.24)	0.215	0.73 (0.40; 1.33)	0.304	0.73 (0.4; 1.33)	0.303	0.96 (0.45; 2.03)	0.916	0.95 (0.44; 2.03)	0.890
Leptotrichia wadei	1.30 (0.69; 2.45)	0.420	0.69 (0.27; 1.76)	0.437	0.66 (0.25; 1.71)	0.391	0.67 (0.27; 1.66)	0.386	0.67 (0.25; 1.83)	0.437	0.62 (0.24; 1.65)	0.340
Mannheimia varigena	0.74 (0.53; 1.04)	0.084	0.85 (0.57; 1.28)	0.446	0.85 (0.56; 1.29)	0.442	0.86 (0.57; 1.29)	0.457	0.88 (0.57; 1.37)	0.581	0.86 (0.55; 1.34)	0.510
Other	1.09 (0.98; 1.22)	0.129	0.96 (0.82; 1.13)	0.644	0.99 (0.84; 1.16)	0.905	0.97 (0.83; 1.14)	0.721	0.96 (0.79; 1.16)	0.657	0.97 (0.80; 1.17)	0.730
Neisseria flavescens	0.50 (0.21; 1.20)	0.121	0.29 (0.06; 1.44)	0.128	0.26 (0.05; 1.35)	0.109	0.39 (0.08; 1.94)	0.248	0.17 (0.02; 1.54)	0.115	0.27 (0.03; 2.48)	0.248
Prevotella histicola	1.99 (0.84; 4.70)	0.117	1.35 (0.43; 4.26)	0.606	1.47 (0.46; 4.69)	0.516	1.38 (0.42; 4.54)	0.595	0.99 (0.27; 3.63)	0.986	0.92 (0.26; 3.29)	0.899
Prevotella maculosa	0.93 (0.19; 4.47)	0.931	0.24 (0.02; 2.93)	0.266	0.29 (0.02; 3.70)	0.343	0.24 (0.02; 3.04)	0.268	0.41 (0.02; 7.27)	0.543	0.46 (0.03; 8.09)	0.596
Prevotella melaninogenica	1.06 (0.86; 1.30)	0.597	1.13 (0.87; 1.46)	0.372	1.11 (0.86; 1.44)	0.424	1.14 (0.88; 1.47)	0.333	1.14 (0.83; 1.56)	0.422	1.14 (0.84; 1.56)	0.403
Prevotella oris	1.64 (0.75; 3.57)	0.216	1.13 (0.47; 2.74)	0.789	1.43 (0.55; 3.74)	0.463	1.17 (0.48; 2.85)	0.732	1.45 (0.47; 4.45)	0.518	1.57 (0.52; 4.68)	0.423
Prevotella oulorum	0.39 (0.13; 1.12)	0.081	0.12 (0.03; 0.54)	0.006	0.12 (0.03; 0.55)	0.007	0.11 (0.02; 0.54)	0.006	0.12 (0.02; 0.62)	0.012	0.15 (0.03; 0.76)	0.023
Prevotella pallens	0.79 (0.36; 1.73)	0.557	0.79 (0.25; 2.49)	0.684	0.89 (0.28; 2.81)	0.847	0.89 (0.29; 2.77)	0.843	1.42 (0.37; 5.44)	0.605	1.50 (0.38; 5.82)	0.562
Prevotella veroralis	1.02 (0.51; 2.04)	0.959	0.45 (0.17; 1.20)	0.110	0.37 (0.13; 1.05)	0.062	0.46 (0.17; 1.23)	0.123	0.30 (0.10; 0.92)	0.035	0.32 (0.11; 0.94)	0.038
Selenomonas noxia	0.74 (0.31; 1.78)	0.508	0.20 (0.06; 0.70)	0.012	0.22 (0.06; 0.77)	0.018	0.19 (0.05; 0.67)	0.010	0.23 (0.06; 0.88)	0.032	0.24 (0.06; 0.93)	0.039
Streptococcus gordonii	0.31 (0.09; 1.15)	0.081	0.19 (0.03; 1.07)	0.060	0.22 (0.04; 1.30)	0.095	0.20 (0.03; 1.13)	0.069	0.27 (0.03; 2.11)	0.211	0.30 (0.04; 2.25)	0.243
Streptococcus mutans	1.00 (0.76; 1.33)	0.978	0.84 (0.38; 1.85)	0.659	0.84 (0.39; 1.81)	0.653	0.84 (0.38; 1.86)	0.664	0.68 (0.25; 1.83)	0.447	0.63 (0.18; 2.18)	0.470

Streptococcus sanguinis	4.58 (1.27; 16.55)	0.020	1.9 (0.70; 5.16)	0.207	2.15 (0.78; 5.90)	0.138	1.86 (0.68; 5.10)	0.228	2.66 (0.87; 8.19)	0.088	2.73 (0.82; 9.11)	0.102
Veillonella alcalescens	1.13 (0.81; 1.59)	0.462	0.93 (0.55; 1.58)	0.800	0.91 (0.55; 1.53)	0.732	0.93 (0.55; 1.57)	0.787	1.18 (0.63; 2.20)	0.613	1.23 (0.65; 2.34)	0.521
Veillonella parvula	0.65 (0.21; 2.04)	0.461	1.26 (0.25; 6.34)	0.782	1.32 (0.27; 6.48)	0.734	1.30 (0.26; 6.47)	0.752	2.53 (0.38; 16.74)	0.335	2.78 (0.41; 18.82)	0.296
Veillonella rogosae	0.12 (0.03; 0.53)	0.005	0.12 (0.02; 0.88)	0.037	0.08 (0.01; 0.69)	0.022	0.11 (0.01; 0.91)	0.041	0.15 (0.01; 1.65)	0.121	0.16 (0.01; 1.78)	0.137

Model 1: crude; Model 2: age, sex, and BMI; Model 3: age, sex, BMI, and bleeding; Model 4: age, sex, BMI, and periodontitis; Model 5: age, sex, BMI, HbA1c, insulin fasting, CRP, and bleeding; Model 6: age, sex, BMI, HbA1c, insulin fasting, CRP, and periodontitis.
